# Predictors of mortality in HIV-1 infected children on antiretroviral therapy in Kenya: a prospective cohort

**DOI:** 10.1186/1471-2431-10-33

**Published:** 2010-05-18

**Authors:** Dalton C Wamalwa, Elizabeth M Obimbo, Carey Farquhar, Barbra A Richardson, Dorothy A Mbori-Ngacha, Irene Inwani, Sara Benki-Nugent, Grace John-Stewart

**Affiliations:** 1 Department of Paediatrics, University of Nairobi, Box 19676 Nairobi 00202, Kenya; 2 Department of Epidemiology University of Washington, 325 Ninth Avenue, Box 359909, Seattle, 98104, USA; 3 Department of Medicine University of Washington, 325 Ninth Avenue, Box 359909, Seattle, 98104, USA; 4 Department of Biostatistics University of Washington, Box 325 Ninth Avenue, 359909, Seattle, 98104, USA; 5 Kenyatta National Hospital, Ngong Road, Nairobi, 00202, Kenya

## Abstract

**Background:**

Among children, early mortality following highly active antiretroviral therapy (HAART) remains high. It is important to define correlates of mortality in order to improve outcome.

**Methods:**

HIV-1-infected children aged 18 months-12 years were followed up at Kenyatta National Hospital, Nairobi after initiating NNRTI-based HAART. Cofactors for mortality were determined using multivariate Cox regression models.

**Results:**

Between August 2004 and November 2008, 149 children were initiated on HAART of whom 135 were followed for a total of 238 child-years (median 21 months) after HAART initiation. Baseline median CD4% was 6.8% and median HIV-1-RNA was 5.98-log_10 _copies/ml. Twenty children (13.4%) died at a median of 35 days post-HAART initiation. Mortality during the entire follow-up period was 8.4 deaths per 100 child-years (46 deaths/100 child-years in first 4 months and 1.0 deaths/100 child-years after 4 months post-HAART initiation). On univariate Cox regression, baseline hemoglobin (Hb) <9 g/dl, weight-for-height z-score (WHZ) < -2, and WHO clinical stage 4 were associated with increased risk of death (Hb <9 g/dl HR 3.00 [95% C.I. 1.21-7.39], p = 0.02, WHZ < -2 HR 3.41 [95% C.I. 1.28-9.08], p = 0.01, and WHO clinical stage 4, HR 3.08 [1.17-8.12], p = 0.02). On multivariate analysis Hb < 9 g/dl remained predictive of mortality after controlling for age, baseline CD4%, WHO clinical stage and weight-for-height z-score (HR 2.95 (95% C.I. 1.04-8.35) p = 0.04).

**Conclusion:**

High early mortality was observed in this cohort of Kenyan children receiving HAART, and low baseline hemoglobin was an independent risk factor for death.

## Background

Sub-Saharan Africa carries the highest burden of paediatric HIV-1 with an estimated 1.8 million children < 15 years infected which represents 90% of all children living with HIV worldwide [1]. In Kenya there are approximately 150,000 HIV-1 infected children, out of whom nearly 60,000 are in need of antiretroviral therapy and about 25,000 are currently accessing treatment [2]. There is a concerted effort to raise the number of children on antiretroviral therapy through increased availability of early infant diagnosis and strengthening provider-initiated counseling and testing in health facilities. As a result, survival of HIV-1 infected children in Kenya and similar settings has dramatically improved as more children access highly active antiretroviral therapy [3-7]. However, mortality within the first few months of starting antiretroviral therapy remains high with various studies reporting between 8% and 15% and most deaths attributable to infections and failure to thrive [3-9]. This level of mortality is substantially higher than what is observed for children initiating HAART in developed nations [10-13].

There is limited but increasing published literature on predictors of early mortality following initiation of HAART and few studies have involved African children. Children with advanced HIV disease manifesting as low weight-for-height, as well as those with very low CD4% have been found to be at highest risk of early mortality following HAART initiation in Cote d'Ivoire, Malawi, and Zambia [4,8,9]. In a large Zambian cohort of children followed up for a limited period of time, younger age and low hemoglobin levels were additional factors associated with higher likelihood of early death following HAART-initiation [8]. Following the results of the Children with HIV Early Antiretroviral Therapy (CHER) Trial in which early treatment reduced early infant mortality by 76%, international guidelines were modified to recommend initiation of HAART for infants below 18 months of age upon HIV diagnosis [14-16]. Initiation of antiretroviral therapy for older children is still dependent on clinical and immunologic staging [15]. Ideally, a child would be diagnosed and treated early, however, in spite of increased efforts to diagnose children early, some don't present for care till they are older. Therefore it remains important to better define factors that impact survival of such children in local settings with a view to maximize benefits by addressing any modifiable cofactors. We describe mortality in a cohort of HIV-1 infected children receiving antiretroviral therapy who have been followed up prospectively since 2004.

## Methods

The Paediatric Adherence Study is a 5-year prospective observational study which started enrolling children in August 2004 as previously described [6]. Children were drawn from the Paediatric wards and HIV-1 Clinic of the Kenyatta National Hospital (KNH) in Nairobi Kenya. The study enrolled children aged 18 months - 12 years who had advanced clinical and/or immunological HIV disease (WHO clinical stage 3-4 or WHO clinical stage 2 with CD4 <15%) and were antiretroviral drug-naïve. After counseling and informed consent, sociodemographic information was obtained and a physical examination performed. Samples were taken for baseline laboratory investigations including full haemogram, T-cell lymphocyte subsets (CD4), plasma HIV-1 RNA, and liver function tests, and a return appointment given for initiation of antiretroviral therapy. Children were initiated on a first-line antiretroviral drug combination recommended by the Kenya national guidelines which consists of two nucleoside reverse transcriptase inhibitors (NRTIs) and one non-nucleoside reverse transcriptase inhibitor (NNRTI) [16]. Follow-up visits were scheduled after 2 weeks, monthly for the first year, and quarterly thereafter. At each follow-up visit, children had a physical examination and information regarding adherence, adverse drug effects, and intercurrent illness was obtained. Hematologic and biochemical tests for toxicity monitoring and plasma HIV-1 RNA were performed 3-monthly during the first year and 6-monthly thereafter, while CD4 counts were obtained every 6 months. Information on the cause of death was abstracted from medical records for children who died in hospital, while for those who died at home, verbal autopsy was used. The caregiver was invited to the clinic to give details of the conditions preceding the child's death and a presumptive diagnosis was reached. In cases where the caregiver was unable to come to the research clinic, the study staff paid a home visit to conduct the verbal autopsy. Written informed consent was obtained from all study participants. Verbal assent was obtained from children between ages 7 and 12 years. This study received ethical approval from the Institutional Review Boards of the University of Washington and the Kenyatta National Hospital.

### Statistical methods

Stata version 8 (Stata Corp, College Station Texas) was used for all analyses. The probability of survival was estimated using the Kaplan-Meier method. Cox proportional hazards models were used to determine baseline characteristics associated with mortality. Factors found to be significant predictors of mortality on univariate Cox proportional Hazards model (p value < 0.05) were entered into a multivariate model. We computed z-scores for anthropometric measures (weight-for-age, height-for-age, and weight-for-height) using the Epi-Info (version 3.2, Centers for Disease Control, Atlanta Georgia) using CDC 2000 reference population. We analyzed weight-for-height z-score as a categorical variable with a cut off value of -2 which is the WHO upper limit for children with moderate protein energy malnutrition [17]. Similarly we categorized hemoglobin at a cut off value of 9 g/dl, below which children in low income countries are considered to have significant iron deficiency anemia [18].

## Results

### Description of study subjects

One hundred and forty nine children initiated HAART between August 2004 and December 2006. Baseline characteristics of the children who initiated HAART are shown in Table 1.

**Table 1:** Baseline characteristics in children who initiated highly active antiretroviral therapy.

Characteristics	Number	Median (IQR) or N (%) (N = 149)
*Socio-demographic*		

Age (years)	147	4.9 (2.6, 6.7)

Female	149	74 (50)

Orphaned Lost one parent	149	44 (30)

Lost both parents	149	10 (7)

*Clinical*		

Hospitalization	147	111 (76)

WHO clinical stage 4	145	20 (14)

Anthropometry		

Weight for age z-score	142	-2.35 (-3.14, -1.49)

Weight-for-height z-score	133	-1.13 (-1.94, -0.21)

Height for age z-score	141	-2.35 (-3.47, -1.22)

*Immunologic*		

CD4 count cells/μl	145	286 (101, 552)

CD4 cell percent	137	6.8 (3.6, 11.4)

CD4 percent severe immune category***	137	122 (89)

Total lymphocyte count cells/mm^3^	144	3762 (2420, 5788)

*Virologic*		

Log_10 _HIV-1 RNA copies/ml	127	5.98 (5.44, 6.46)

Hemoglobin g/dl	146	10.2 (9.0, 11.6)

Serum albumin	137	33.0 (28.8, 39.0)

*** Age specific WHO categories for HIV infected children (33)

### Follow-up and outcomes

**Figure 1:**
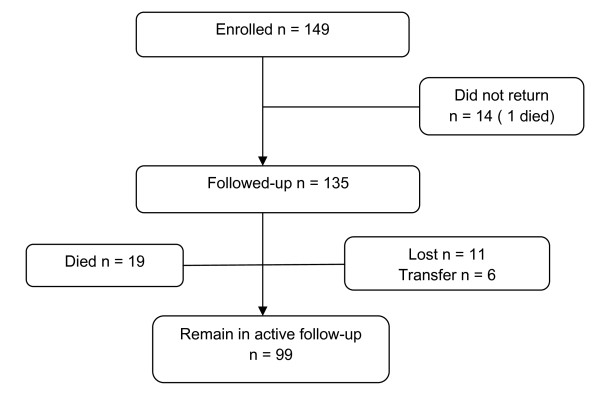
**Follow-up and retention in the cohort**.

Figure 1 provides a summary of follow-up and mortality among these children. Out of the 149 children who initiated HAART, 135 returned for at least one scheduled follow-up visit two weeks after initiation of treatment. Of the 14 children who did not complete any clinic follow-up, one child died 2 days after HAART initiation while for 13 (9%) children there was no follow-up information available after initiating antiretroviral therapy. By October 2008, median follow-up time on antiretroviral therapy for the 135 who returned to clinic was 21 months (IQR 6, 33) equivalent to 238 child-years. While on follow-up, six (4%) children were transferred to other medical facilities on request of caregivers and 20 (13%) children died. Eleven children (7%) were lost to follow-up after completing at least one clinic visit after HAART initiation.

### Mortality

**Figure 2:**
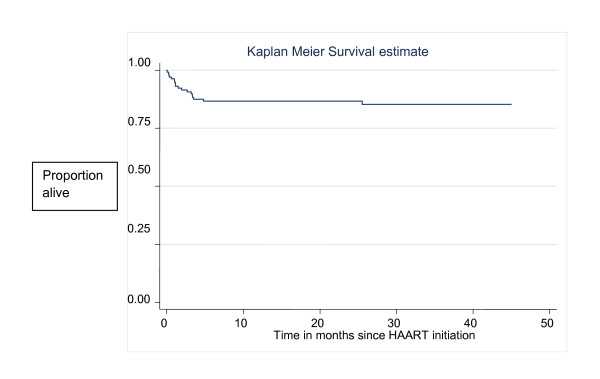
**Probability of survival among children receiving HAART**.

Twenty children died at a median of 35 days post HAART initiation (IQR 13 - 99 days), of whom 18 (90%) died in the first 120 days. The mortality rate over the entire follow-up period was 8.4 deaths per 100 child-years (20 deaths over 238 child-years). Mortality in the first 4 months of follow-up was 46 deaths per 100 child-years (18 deaths over 39 child-years) but this dropped to 1.0 death per 100 child-years between 4 months and 2 years post-HAART (2 deaths over 199 child-years). The cumulative survival was 95% after 1 month of HAART, 89% after 3 months and 85% after 6 and 12 months respectively. The cumulative survival after 25 months was 84% (Figure 2).

### Correlates of mortality

On univariate analysis, low baseline hemoglobin level (<9 g/dl), low baseline weight-for-height z-scores < -2, and WHO stage 4 at baseline were significantly associated with mortality (Table 2). Trends for association with mortality were also observed for prior hospitalization, baseline total lymphocyte count, baseline CD4 count, and serum albumin.

**Table 2:** Correlates of mortality among children who initiated HAART.

Univariate					
***Characteristic***	***HR***	***(95% CI)***	***p***	***Number***	***Percentage*** ***(out of 149)***

***Clinical, sociodemographic***					

Age ≥3 yrs)	1.30	(0.47-3.62)	0.6	147	99

Sex (female)	0.79	0.33 - 1.91	0.6	149	100

WHO stage 4	3.08	1.17-8.13	**0.02**	145	97

Hospitalized since birth	7.09	0.95-53.14	0.06	147	97

Weight-for-height z score < -2	3.41	1.28-9.08	**0.01**	142	95

Tuberculosis at baseline	1.49	0.62-3.57	0.4	149	100

Lost parent	1.25	0.50-3.13	0.6	149	100

*Laboratory*					

Log HIV-1 RNA	0.80	0.58-1.11	0.2	127	85

CD4 count (per 100 cells)	0.86	0.73-1.02	0.09	145	97

CD4 percent < 15%	1.74	0.40-7.56	0.5	137	92

Hemoglobin (< 9 g/dl)	3.00	1.21-7.39	**0.02**	146	98

Total lymphocyte count (per 1000)	0.79	0.63 - 1.00	0.05	144	97

Serum albumin	0.93	0.86-1.00	0.05	137	92

**Multivariate**					

Weight-for-height z-score < -2	2.48	0.87-7.04	0.09	142	95

Hemoglobin (<9 g/dl)	2.95	1.04-8.35	**0.04**	146	98

WHO stage 4	3.03	0.96-9.55	0.06	147	99

### Multivariate analysis

When the significant variables, hemoglobin < 9 g/dl and baseline weight-for-height z-score < -2, and WHO clinical stage 4 were entered into a single Cox proportional Hazards model, hemoglobin < 9 g/dl remained predictive of mortality (HR 2.95 [95% C.I. 1.04, 8.35], p = 0.04). There were trends of association between weight-for-height z-score < -2 and mortality (HR 2.48 [95% C.I. 0.87, 7.04], p = 0.09) as well as WHO clinical stage 4 and mortality (HR 3.03 [95% C.I. 0.96-9.55], p = 0.06), Table 2.

### Causes of mortality

Nineteen of the 20 (95%) children who died had been hospitalized at least once since birth and 18 of them were recruited into the study from the Kenyatta National Hospital paediatric wards. Nine of 20 (45%) children died in health facilities while 11 (55%) died at home. Of those dying in health facilities, 8 died in Kenyatta National Hospital while 1 child died in the local district hospital. Eleven (55%) children had evidence of pneumonia either from hospital records or information given through verbal autopsy at the time of death. Five children (25%) had congestive heart failure with ventricular dysfunction secondary to possible HIV associated cardiac disease. One of these children had dilated cardiomyopathy on echocardiography. Tuberculosis contributed to death in 4 children (20%) and one child died from each of the following conditions: acute diarrhea, Non-Hodgkin's lymphoma, and anemia. The primary cause of death could not be established for one child who died at home. Additional file 1, Table S3 summarizes the characteristics of the 20 children who died after initiation of antiretroviral therapy.

## Discussion

In this cohort of HIV-1 infected children who initiated highly active antiretroviral therapy at advanced immunosuppression we observed high early mortality. The first 4 months after HAART initiation were associated with highest mortality, and children who survived this period were less likely to die in the subsequent 2 years of follow-up. In our cohort pre-treatment hemoglobin <9 g/dl, weight-for-height z-scores < -2, and WHO clinical stage 4 were associated with mortality on univariate analysis. Only hemoglobin < 9 g/dl remained significantly predictive of mortality on multivariate analysis. However, with 20 events, the power for assessing multiple covariates was limited and trends remained for weight-for-height z-score < -2 and WHO clinical stage 4.

Thirteen percent of the children died over the entire period of follow-up, 12% within the first 4 months of starting HAART. This falls within the range of mortality observed in similar African cohorts, but significantly higher than death rates among HAART-treated children in industrialized countries [3,4,8-12]. A recent review comparing mortality in HIV-1 infected children receiving HAART between resource-rich and resource-limited settings found mean mortality rates of 2.9 vs. 9.0 deaths per 100 person years, respectively, or 4.4% vs. 8.1% with higher death rates in poor countries [13]. The main differences identified between children initiating HAART in the two settings were higher baseline CD4 levels (24% vs. 11%) and lower baseline viral loads (4.87 log vs. 5.57 log copies/ml) in resource-rich countries compared to resource-poor settings, respectively. These differences indicate that children in resource-rich settings generally initiated HAART earlier in their illness and suggest that early initiation of HAART will most likely result in fewer deaths. The difference in mortality rates could also be due to the limited supportive care available to children in resource-poor settings at the most crucial period before they immune reconstitute to a level that reduces their vulnerability to potentially fatal opportunistic infections. Finally, the disparate mortality rates may reflect a difference in the rates of Immune reconstitution inflammatory syndrome (IRIS) between the two settings. One child in our study died from probable TB - IRIS and it is possible that IRIS contributed to other deaths.

The pattern of mortality we observed is typical of what has been described in other cohorts of HIV-1 infected African children receiving HAART [8,9]. The death rate dropped from a high of 46 deaths per 100 person-years to 1 death per 100 person years, a remarkable fall after the 4-month post-HAART initiation point. The consistent pattern of high mortality in the first 3-6 months before a dramatic and sustained decrease in death rate suggests that children with severe immune deficiency require some time before the benefits of HAART are fully realized. It appears that with the current approach to therapy it may be difficult to salvage some children with extremely advanced HIV and the number of such children increases as HAART is initiated much later in the disease course.

Although there are no data for age-specific mortality figures for Kenyan children above 5 years, it is notable that the mortality in our study, (13%) approximates the current under-5 child mortality in Kenya (128 per 1000 live births) [19]. In contrast, mortality approaching 50% was reported in an untreated cohort of HIV-1 infected Kenyan children [20]. Thus the use of HAART has substantially reduced mortality to rates that are at least as high as those observed in the general population compared to the pre-HAART period when mortality was about 3 times higher in HIV infected children.

Our finding of anemia as a predictor of mortality is consistent with reports from few paediatric and adult African cohorts [8,9]. It is notable that the levels of anemia in question (Hb < 9 g/dl) are modest and would not normally lead to rapid clinical deterioration. It is more likely that anemia is a surrogate marker for advanced HIV [21,22]. In addition, anemia is a well recognized component of severe protein-energy malnutrition which exists in 5-10% of Kenyan children [23]. We also found a trend of association between low weight-for-height and mortality. Growth failure (measured as low weight-for-height or low weight-for-age) has been found to independently predict mortality in several studies [8,9,24]. In the context of our study, in which 89% children were classified in the severe immune category, weight-for-height z-score < -2 may represent additional nutritional deprivation. The effect of severe protein energy malnutrition on both humoral and cell-mediated immunity has been previously described [25-27]. It is conceivable that children with both conditions, advanced HIV -1 disease and severe protein energy malnutrition will have limited capacity for immune recovery and are especially prone to life-threatening microbial infections.

Unlike several paediatric studies, we did not demonstrate a significant association between the baseline CD4% or viral load and mortality [7,8,28]. This may be due to the small sample size and generally low CD4 counts, thus limiting power to detect differences between various CD4 thresholds.

Infectious conditions, specifically pneumonia, contributed to the greatest number of deaths followed by underlying cardiac conditions and tuberculosis. Although infections remain an important cause of mortality in HAART-treated children in Western cohorts, the proportion and overall burden of infectious illness is less [12]. Given such a high burden of infectious illness in this cohort, it is also possible that IRIS played a role in some of the deaths. A large proportion of the children had known risk factors for IRIS such as low weight-for-age and severe immune suppression [29]. Cardiac-related causes contributed to approximately a quarter of the deaths in our study. HIV-associated cardiac involvement presenting as myocarditis, left ventricular dysfunction, and dilated cardiomoypathy has been described in patients with advanced disease with low CD4 counts such as the majority of the children in our cohort [30]. The mechanisms for cardiac disease include direct effect of HIV, other cardiotropic viruses, cytokines and opportunistic infections [31,32].

Strengths of our study include long follow-up of children starting HAART with data on many concurrently important predictors of mortality (including viral load, CD4% and nutritional parameters). Limitations of our study include the relatively small sample size, the lack of post-mortem studies, and for children who died at home, reliance on verbal autopsy to determine the cause of death. This may lead to inaccurate labeling of the cause of death in such cases. In addition the substantial number of children lost to follow-up may include children who died and thereby our study may underestimate mortality.

The importance of our findings relate to building evidence that it may be possible to identify the children at highest risk of early death following initiation of HAART in resource-poor settings. Efforts aimed at prompt identification of HIV infected infants early in the disease course should be prioritized to enable timely initiation of HAART in order for children to realize maximal benefits from expanding treatment programs. It is also important to investigate targeted interventions that intensify support for children during the first 4-6 months following HAART initiation with particular attention given to those presenting with anemia and low weight-for-height.

## Conclusion

Weight-for-height z-score < -2, Hemoglobin <9 g/dl and WHO stage IV were predictive of mortality on univariate analysis. Only Hemoglobin <9 g/dl at baseline remained independently predictive of mortality. Besides efforts to initiate HAART earlier in the course of illness, HIV infected children presenting with anemia and severe wasting should be especially prioritized for care post-HAART initiation. Targeted research on the role of nutritional support and correction of anemia should be considered.

## Competing interests

The authors declare that they have no competing interests.

## Authors' contributions

All authors read and approved the final manuscript. DW was the lead author who led the design and conduct of the study as well as manuscript development. EO provided clinical expertise and epidemiological input in manuscript development. CF provided epidemiological support for manuscript development. BAR provided guidance on study design and led statistical analysis. DMN gave input on clinical and epidemiological aspects of the study and manuscript development. II provided clinical care of the children in study and gave input on clinical aspects of manuscript development. B provided statistical support while GJS provided mentorship in the overall design and epidemiological aspects of the study and manuscript development.

## Pre-publication history

The pre-publication history for this paper can be accessed here:

http://www.biomedcentral.com/1471-2431/10/33/prepub

## Supplementary Material

Additional file 1 **Table S3. Characteristics of children who died after initiation of highly active antiretroviral therapy**. This table contains a detailed description of all the children who died following initiation of antiretroviral therapy including their ages at enrolment, baseline CD4 and plasma HIV-1 RNA, presenting diagnosis, duration on antiretroviral therapy and suspected cause of death. Click here for file
