# Supplementary material for: Predictors of mortality in HIV-1 infected children on antiretroviral therapy in Kenya: a prospective cohort
Source: BMC Pediatr. 2010 May 18;10:33. doi: 10.1186/1471-2431-10-33 (PMC2887829; doi:10.1186/1471-2431-10-33)
Supplement: Additional file 1 — Table S3. Characteristics of children who died after initiation of highly active antiretroviral therapy. This table contains a detailed description of all the children who died following initiation of antiretroviral therapy including their ages at enrolment, baseline CD4 and plasma HIV-1 RNA, presenting diagnosis, duration on antiretroviral therapy and suspected cause of death. [file 1471-2431-10-33-S1.DOC]

Table S3. Characteristics of children who died after initiation of highly active antiretroviral therapy.

| Child | Age (yrs) | Baseline CD4% | Baseline Hb g/dl | Baseline viral load | Presenting Diagnosis | Days on HAART | Venue and Cause of death |
| --- | --- | --- | --- | --- | --- | --- | --- |
| 1 | 10 | 5.1 | 7.2 | 6.95 | Tuberculosis | 105 | District hospital disseminated TB /IRIS |
| 2 | 1.8 | 6.4 | 11.2 | 6.99 | FTT, pneumonia, HIV encephalopathy | 101 | KNH Severe pneumonia, Septicemia |
| 3 | 6.5 | 5.7 | 11.0 | 5.41 | Cor pulmonale | 35 | KNH pneumonia, HIV related ventricular dysfunction |
| 4 | 4.3 | 1.7 | 8.30 | 5.80 | Pneumonia Cor pulmonale | 34 | Home Severe Pneumonia, heart failure |
| 5 | 4.8 | 3.2 | 8.2 | 6.95 | Pneumonia Cor pulmonale | 27 | Home pneumonia, HIV related ventricular dysfunction |
| 6 | 7.9 | 8.4 | 8.3 | 6.95 | Nodular Kaposi’s sarcoma | 100 | KNH Acute diarrhea |
| 7 | 8.2 | 18.6 | 7.5 | 1.0 | Non Hodgkin’s lymphoma | 10 | KNH Non-Hodgkins lymphoma, Heart failure |
| 8 | 8.2 | 1.3 | 10.8 | 5.64 | PTB, Cor pulmonale | 45 | Home TB, HIV related ventricular dysfunction |
| 9 | 7.4 | 9.8 | 11.1 | 5.95 | Pneumonia | 14 | Home Unknown |
| 10 | 5.7 | 1.6 | 10 | 6.48 | Pneumonia Chronic diarrhea, Tuberculosis | 70 | Home Pulmonary Tuberculosis |
| 11 | 1.5 | 1.4 | 10.2 | - | Meningitis, TB, HIV encephalopathy | 36 | KNH Pneumonia, Tuberculosis |
| 12 | 7.2 | 12.4 | 7.3 | 5.06 | Anaemia | 8 | KNH Anemia |
| 13 | 3.3 | 8 | 8 | - | Lymphadenopathy | 85 | Home Pneumonia |
| 14 | 1.5 | 14.1 | 12 | 5.93 | Cor Pulmonale | 30 | Home Pneumonia , HIV related ventricular dysfunction |
| 15 | 1.5 | 9.8 | 9 | 5.43 | Pneumonia | 7 | KNH Severe Pneumonia |
| 16 | 6 | 1.7 | 4.9 | 4.65 | Pneumonia | 144 | Home Severe Pneumonia |
| 17 | 8.6 | 19.1 | 9.9 | 4.63 | Pneumonia | 34 | KNH Dilated cardiomyopathy |
| 18 | 6.3 | 12.7 | 9.5 | 5.29 | Pneumonia | 18 | Home Severe Pneumonia |
| 19 | 9.3 | 4.2 | 7.7 | 6.24 | Pneumonia | 96 | Home Unknown |
| 20 | 2 | - | - | - | Pulmonary Tuberculosis | 2 | Home Severe Pneumonia |
